# Assay optimisation and technology transfer for multi-site immuno-monitoring in vaccine trials

**DOI:** 10.1371/journal.pone.0184391

**Published:** 2017-10-11

**Authors:** Steven G. Smith, Stephanie A. Harris, Iman Satti, Donna Bryan, K. Barry Walker, Hazel M. Dockrell, Helen McShane, Mei Mei Ho

**Affiliations:** 1 Department of Immunology and Infection, Faculty of Infectious and Tropical Diseases, London School of Hygiene and Tropical Medicine, London, United Kingdom; 2 The Jenner Institute, Oxford, United Kingdom; 3 Bacteriology Division, Medicines and Healthcare products Regulatory Agency-National Institute for Biological Standards and Controls, South Mimms, Potters Bar, Hertfordshire, United Kingdom; Fundació Institut d’Investigació en Ciències de la Salut Germans Trias i Pujol, Universitat Autònoma de Barcelona, SPAIN

## Abstract

Cellular immunological assays are important tools for the monitoring of responses to T-cell-inducing vaccine candidates. As these bioassays are often technically complex and require considerable experience, careful technology transfer between laboratories is critical if high quality, reproducible data that allows comparison between sites, is to be generated. The aim of this study, funded by the European Union Framework Program 7-funded TRANSVAC project, was to optimise Standard Operating Procedures and the technology transfer process to maximise the reproducibility of three bioassays for interferon-gamma responses: enzyme-linked immunosorbent assay (ELISA), ex-vivo enzyme-linked immunospot and intracellular cytokine staining. We found that the initial variability in results generated across three different laboratories reduced following a combination of Standard Operating Procedure harmonisation and the undertaking of side-by-side training sessions in which assay operators performed each assay in the presence of an assay ‘lead’ operator. Mean inter-site coefficients of variance reduced following this training session when compared with the pre-training values, most notably for the ELISA assay. There was a trend for increased inter-site variability at lower response magnitudes for the ELISA and intracellular cytokine staining assays. In conclusion, we recommend that on-site operator training is an essential component of the assay technology transfer process and combined with harmonised Standard Operating Procedures will improve the quality, reproducibility and comparability of data produced across different laboratories. These data may be helpful in ongoing discussions of the potential risk/benefit of centralised immunological assay strategies for large clinical trials versus decentralised units.

## Introduction

Immune bioassays are essential tools with which to measure the immunogenicity of vaccines. Some of these are well established and can indicate the presence or absence of protection in vaccinated individuals where the assay detects a reliable correlate of protection such as neutralising antibody [1]. However, bioassays used to determine the immunogenicity of T-cell-inducing vaccines are often more complex and require a greater degree of operator expertise and experience. There are many T-cell effector mechanisms such as surface receptor upregulation, cytokine secretion, proliferative capacity, degranulation and cytotoxic capabilities that may be measured. Most T-cell assays require an antigen re-stimulation step, which is necessary to detect vaccine-specific responses that are recognised as often representing only a small fraction of the total T-cell compartment [2]. Some T-cell assays can simultaneously measure more than one parameter of interest such as responder cell phenotype together with cytokine secretion profile using flow cytometric assays or multiple secreted effector molecules using multiplex bead array or dual/triple colour enzyme-linked immunospot (ELISpot). Others only measure one effector function (e.g. single cytokine ELISpot or enzyme-linked immunosorbent assay (ELISA) for interferon-gamma (IFNγ)).

With a wide array of immunological bioassays available, as well as numerous potential modifications depending on the focus of a study, it is not surprising that a review of the literature reveals various bioassays in use, even for vaccines against a single pathogen [3–10]. There are advantages to a more consistent and co-ordinated approach to cellular immuno-monitoring within candidate vaccine trials, particularly to facilitate inter-site comparisons of the same and different candidate vaccines and to reduce the inherent variability in data generated by these complex assays. There is also an ongoing discussion of the merits and risks associated with centralising immune bioassays to one laboratory, versus the complexity of comparing data from multiple laboratory sites, this study may provide data to aid those discussions.

TRANSVAC was a European Union, Framework Program 7-funded consortium project with the aim of accelerating the development of promising vaccine candidates by developing, optimising and standardising state-of-the-art processes and facilities available to vaccine developers to bridge the gap between bench research and clinical assessment of novel vaccines [11]. We describe here our efforts to optimise and standardise one of these processes, namely the monitoring of vaccine immunogenicity using bioassays. We compared three cellular immunological assays in three participating laboratories: an ELISpot assay; an intracellular cytokine staining (ICS) assay and an ELISA assay, each designed to measure antigen-specific IFNγ. These assays are three of the most important and commonly used assays in pre-clinical studies and clinical trials of vaccines for tuberculosis, malaria and Human Immunodeficiency Virus [2,12–14]. The project focussed on standard operating procedure (SOP) transfer and assay establishment, followed by harmonisation, and assessment of performance and reproducibility between groups. Critically, common reference standards were shared among participating laboratories to enable optimisation to be better assessed, including IFNγ protein standard for ELISA and cryopreserved donor peripheral blood mononuclear cells (PBMC) for all experiments. This manuscript describes the role of SOP harmonisation and operator training in improving inter-site reproducibility of assay performance.

## Materials and methods

### Study design

The main aim of the study was to compare the performance of three cellular immunological assays across three different laboratories. The objectives were first to assess the results obtained using the assays when SOPs were shared between participating laboratories in their existing format, and second to investigate what measures could be employed to improve the reproducibility of each assay. The study therefore comprised a series of three experimental “rounds”. Operators performed round one assays in their own laboratory setting following a straightforward sharing of agreed assay SOPs and cryopreserved PBMC aliquots from healthy adult buffy coats. Round two consisted of side-by-side training sessions during which operators performed assays together in the lead laboratory for each assay to identify variability in technique and practice not captured by the SOPs. Round three assays took place following training sessions and involved operators performing assays individually in their own laboratories. In order to strengthen the assessment of improvements in assay performance, aliquots of the PBMC samples used in round one were used again in round three.

### Donors

PBMC were chosen as the tissue for investigation as these are more easily cryopreserved and shared between groups. Heparinised peripheral blood samples were either obtained from healthy adult donors (for some inter-group “side-by-side” comparisons) or from local blood transfusion services as buffy coats (all other inter-group comparisons). PBMC from these samples were cryopreserved at one laboratory and equal numbers of vials distributed to the other two laboratories. The Ethics Committee of the London School of Hygiene and Tropical Medicine (ref. 5520) and the NHS Berkshire Research Ethics Committee (REC ref. 06/Q1602/146) gave ethical approval for the use of these samples. Informed, written consent was obtained from adults who donated blood directly to the study.

### Sample processing

PBMC were isolated from blood samples and cryopreserved in aliquots. Briefly, following isolation from whole blood by density centrifugation, PBMC were counted and re-suspended in foetal bovine serum (FBS). Cells were then chilled for 30 minutes (min) on ice after which an equal volume of chilled FBS containing 20% dimethylsulfoxide was slowly added. Cells were distributed into Cryovials (Nunc) at 5x10^6^ cells per tube and frozen overnight at −80°C in Mr. Frosty containers (Nalgene) before transfer to liquid nitrogen. Prior to use, an appropriate number of cryovials were defrosted by each operator. Vials were thawed in a 37°C water bath until only a small bead of ice remained. The contents were then transferred to a pre-prepared centrifuge tube containing R10 assay medium (RPMI 1640; 10% FBS; 2 mM L-glutamine; 1% penicillin/streptomycin; 1% sodium pyruvate, all from Sigma), centrifuged then re-suspended in R10 containing 10 units per ml of Benzonase (Novogen). Cells were then rested at 37°C for 2 hours (h) prior to use.

### ELISA

IFNγ ELISAs were performed on supernatants generated in PBMC stimulation assays. PBMC prepared as described above were incubated in R10 medium at 2x10^5^ cells per well in U-bottomed, 96-well plates (Costar). Antigens were added to achieve a final volume of 200 μl per well and at concentrations of 10 μg/ml *Mycobacterium tuberculosis* purified protein derivative (PPD for *in vitro* use, batch RT50; Statens Serum Institute) and 5 μg/ml phytohaemagglutinin (PHA; Sigma). Medium alone was used as a negative control. After incubation for 72 h at 37°C, supernatants were harvested and stored at −80°C for ELISA analysis. ELISA plates were coated overnight at 4°C with 2 μg/ml anti-IFNγ capture antibody (BD Biosciences), washed (phosphate-buffered saline (PBS) with 0.05% Tween 20) and blocked with PBS containing 10% FBS for 2 h at room temperature. After a further wash, 50 μl of PBMC assay supernatant, either undiluted or diluted to 1/3, 1/9 or 1/27, was added to wells in duplicate as was 50 μl of IFNγ protein standard (BD Biosciences) in doubling dilutions from 4000 pg/ml to 31.25 pg/ml and 50 μl of IFNγ positive control supernatant. Plates were incubated overnight at 4°C, washed and probed with anti-IFNγ-biotin (BD Biosciences), Avidin-Peroxidase (Sigma, UK) and finally OPD Fast solution (Sigma, UK) for colour development. Plates were read at 490nm. ELISA IFNγ concentrations were estimated from linear standard curves generated in Microsoft Excel by plotting optical density readings against standard concentrations. As described previously for this ELISA method [15], the upper and lower limits of detection corresponded to the highest and lowest standard curve values (4000 pg/ml and 32 pg/ml respectively) although in this manuscript concentrations above 4000 pg/ml have been obtained and reported following extrapolation from the standard curve. Values below the lower limit of detection were given the value of 15.5 pg/ml (half the lower limit of detection).

### ELISpot assay

ELISpot plates were pre-coated overnight at 4°C with 15 μg/ml of anti-IFNγ coating antibody (MabTech, Sweden) followed by washing and blocking for 2–5 h in R10 medium. PBMC were prepared as described above and finally re-suspended at 3.75x10^6^ cells per ml in R10. The blocking solution was removed and PBMC added to ELISpot wells at 3x10^5^ PBMC per well in 80 μl of R10. Stimuli and controls were prepared at appropriate concentrations in R10 and 20 μl of each stimulus added to wells in replicates of 6 (Final concentrations– 20 μg/ml PPD; 1 pg/ml Staphylococcus Enterotoxin B (SEB); 6.25 μg/ml FEC peptides (pool of 32 peptides from Flu/EBV/CMV)). For samples from donors who on previous occasions had displayed strong responses that produced too many spots to count, PBMC were added at half the usual number (1.5x10^5^ per well) for certain stimuli to achieve countable spot numbers. ELISpot plates were incubated overnight at 37°C then washed with PBS with 0.05% Tween 20. A biotinylated anti-IFNγ detection antibody (Mabtech, Sweden) was added to all wells at 1 μg/ml for 2 h at room temperature. Plates were washed again and streptavidin-alkaline phosphatase reagent (Mabtech, Sweden) added for 1 h at room temperature. After a final wash, plates were developed with NBT/NCIP reagent until spots were visible. The reaction was stopped by washing plates with tap water. After drying overnight, spots were enumerated using an automated ELISpot reader (AID version 5.0). The same ELISpot reader, software and count settings were used at all sites and ELISpot results were reported in spot-forming cells (SFC) per million PBMC.

### ICS assay

PBMC were prepared as described above, re-suspended in R10 medium at 10^6^ cells per ml and distributed into 5 ml FACs tubes (Invitrogen, UK) at 10^6^ cells per tube; one tube per stimulation condition. Stimuli were added to each tube as appropriate (R10 medium as a negative control; PPD at 20 μg/ml; SEB at 5 μg/ml; FEC peptides at 25 μg/ml) and samples were incubated at 37°C for 2 h. After this time, 3 μl of brefeldin A (BFA, Sigma, UK; stock concentration 1 mg/ml) was added to all tubes to give a final concentration of 3 μg/ml, and tubes were incubated for a further 18 h (overnight) at 37°C. Following stimulation, PBMC were washed in FACS buffer (PBS with 0.1% bovine serum albumin (Sigma) and 0.01% sodium azide (Sigma)) and stained with VIVID live/dead reagent (Molecular Probes) as well as with a surface stain cocktail of antibodies (anti-CD4-APC-Cy7 (Biolegend); anti-CD14-Pacific Blue (Invitrogen); anti-CD19-Pacific Blue (eBiosciences)). After further washing, PBMC were permeabilised with Cytofix/Cytoperm reagent (BD Biosciences) and stained with an intracellular antibody cocktail (anti-CD3-PerCP (Biolegend); anti-CD8-FITC (Biolegend); anti-IFNγ-PE (Caltag)) prior to a final wash and re-suspension in 1% paraformaldehyde. Cells were acquired within 24 h of staining.

### Data analysis

Flow cytometric analysis was performed using FlowJo software (Treestar). Gating was performed using a previously published gating strategy [16]. For all assays, averages were calculated from replicates of each condition and background measurements from negative controls were subtracted to give final data points using Microsoft Excel. Background data (negative control data measured in all assays, for each round and for each site) are presented as supplementary data (S1 Tables). Background corrected data was plotted in Prism 7.0 (GraphPad) and Microsoft Excel. In order to allow site-to-site assay variability to be determined by coefficients of variation (CVs), mean and standard deviation of responses measured at all 3 sites were calculated in Microsoft Excel and are presented as summary statistics. Pre- and post-training inter-site coefficients of variation (CVs) were compared using Mann-Whitney U tests. Logarithmic regression analysis to determine the relationship between inter-site CVs and magnitude of response measured for each assay was performed in Microsoft Excel.

## Results

### Initial inter-site assay comparisons using shared protocols, reagents and stimuli

Following an initial review of different versions of assay SOPs used in each participating laboratory, we found that SOPs for the same assay varied considerably across the groups. Therefore, the optimal version of each SOP was selected and shared between sites for all further experiments. Reagents and stimuli were also ordered centrally and shared.

**Fig 1:**
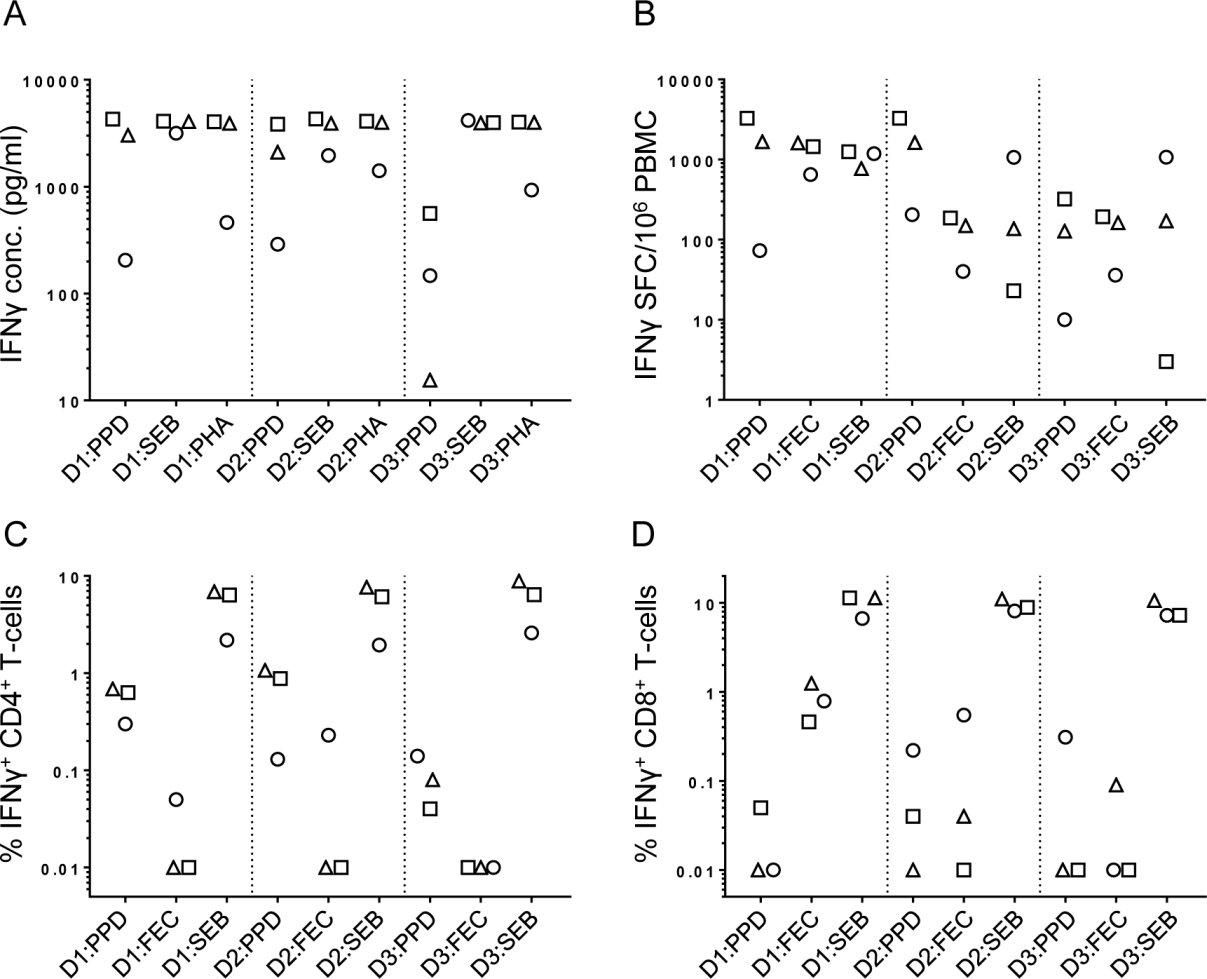
Initial inter-site performance of assays of cellular IFNγ response. Assays measuring IFNγ responses in stimulated PBMC were performed at three laboratory sites using shared PBMC aliquots from three donors (D1-D3) and the indicated antigens/mitogens. Figure panels display responses measured in each assay: ELISA (A), ELISpot (B), CD4+ T-cell ICS (C) and CD8+ T-cell ICS (D) and symbols indicate different sites: Site 1 (squares), Site 2 (triangles), Site 3 (circles). All response measurements displayed have been corrected for background.

A common set of frozen PBMC from three donors was used to test immune responses at three sites. ELISA, ELISpot, CD4+ T-cell ICS and CD8+ T-cell ICS assay results are shown in Fig 1A–1D. Mean assay responses across sites and assay CV data are shown in Table 1. Despite the use of identical SOPs, there were still notable variations in the responses to the antigen stimuli PPD (for all assays) and FEC (for ELISpot and ICS assays). Sites were more consistent in their measurements of strong responses to the positive controls (SEB and PHA) although this varied across different assays. For example, the ELISpot assay demonstrated a greater variation in response to SEB. In general, there was more variation across sites when measured responses were weak. This was particularly noticeable in situations where an antigen stimulus was sub-optimal for the induction of a response, e.g. FEC-induced CD4+ T-cell and PPD-induced CD8+ T-cell responses.

**Table 1:** Variation in IFNγ responses measured across 3 sites.

Assay	Donor	Antigen	Site 1	Site 2	Site 3	Mean response (SD)	CV (%)
ELISA (pg/ml)	A	PPD	4288	3029	205	2507 (2091)	83
		SEB	4089	4057	3165	3770 (525)	14
		PHA	4041	3910	462	2804 (2030)	72
	B	PPD	3834	2106	289	2077 (1773)	85
		SEB	4295	3907	1957	3386 (1253)	37
		PHA	4089	3984	1409	3161 (1518)	48
	C	PPD	564	16	147	242 (286)	118
		SEB	3967	3946	4169	4027 (123)	3
		PHA	4016	3979	931	2975 (1771)	60
ELISpot (SFC)	A	PPD	3282	1667	73	1674 (1605)	96
		FEC	1449	1607	649	1235 (514)	42
		SEB	1254	772	1182	1069 (260)	24
	B	PPD	3269	1628	205	1701 (1533)	90
		FEC	186	149	40	125 (76)	61
		SEB	23	137	1065	408 (572)	140
	C	PPD	321	128	10	153 (157)	103
		FEC	193	163	36	131 (83)	64
		SEB	3	171	1069	415 (573)	138
CD4 ICS (%IFNγ+)	A	PPD	0.63	0.69	0.30	0.54 (0.21)	39
		FEC	0.01	0.00	0.05	0.02 (0.03)	140
		SEB	6.37	6.88	2.19	5.15 (2.58)	50
	B	PPD	0.88	1.07	0.13	0.69 (0.49)	71
		FEC	0.00	0.00	0.23	0.08 (0.13)	168
		SEB	6.11	7.64	1.95	5.23 (2.94)	56
	C	PPD	0.04	0.08	0.14	0.08 (0.05)	57
		FEC	0.00	0.01	0.00	0.003 (0.004)	131
		SEB	6.41	8.84	2.59	5.95 (3.15)	53
CD8 ICS (%IFNγ+)	A	PPD	0.05	0.00	0.01	0.02 (0.03)	145
		FEC	0.46	1.25	0.79	0.83 (0.4)	48
		SEB	11.39	11.39	6.70	9.83 (2.7)	28
	B	PPD	0.04	0.00	0.22	0.09 (0.12)	135
		FEC	0.00	0.04	0.55	0.2 (0.3)	154
		SEB	8.90	11.04	8.14	9.36 (1.5)	16
	C	PPD	0.00	0.00	0.31	0.1 (0.18)	174
		FEC	0.00	0.09	0.00	0.03 (0.05)	173
		SEB	7.25	10.64	7.24	8.37 (1.96)	23

Three assays, three blood donors and three antigens/positive control stimulants were used in each laboratory site to compare measured immune responses

### Side-by side, operator training eliminates minor variations in approach between sites

The next step taken to eliminate inter-site variation was an “operator training” session. Although operators at each site possessed considerable expertise and experience in performing the assays involved, it was agreed that variations in each operator’s training, differences in SOP interpretation, “common practice” in a given laboratory and previous experience might lead to measurable differences when two operators’ data were compared. To counter this, each assay was performed in a side-by-side arrangement where one operator acted as curator for a given assay and trained the other operators in the specific individual approach of that operator to further harmonise the technique of operators when acting alone.

**Fig 2:**
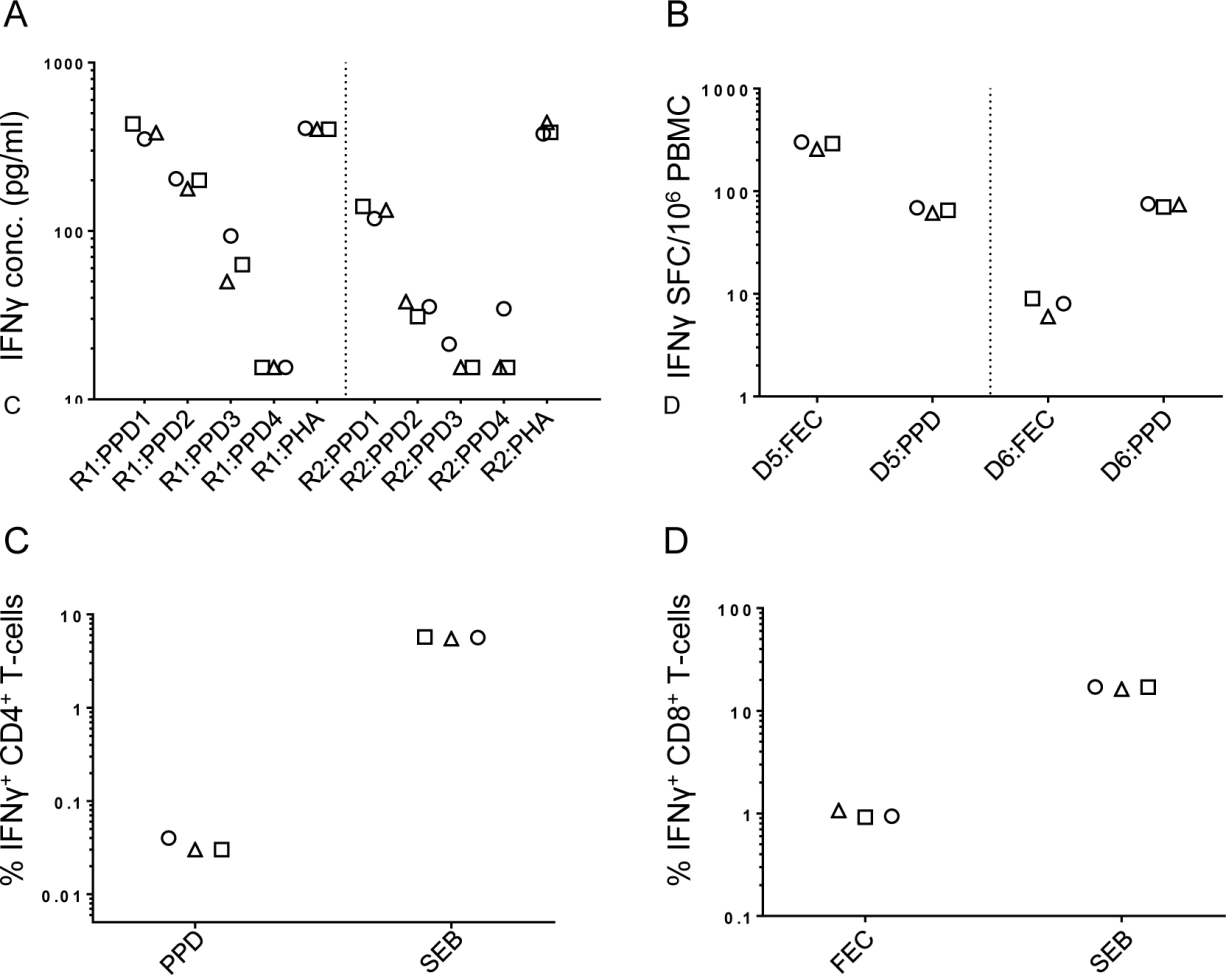
Assay responses measured during joint, inter-site training sessions. ELISA (A), ELISpot (B), CD4+ T-cell ICS (C) and CD8+ T-cell ICS (D) assays were each performed by operators from the three participating sites in side-by-side training sessions: Site 1 (squares), Site 2 (triangles), Site 3 (circles). For ELISA measurements (A), assays were performed on serially diluted supernatants from PPD-stimulated samples: PPD1, undiluted; PPD2, 1 in 3 diluted; PPD3, 1 in 9 diluted; PPD4, 1 in 27 diluted. All assays were performed on PBMC from different, locally sourced donors. ELISPot assays were performed on 2 donors (D5 and D6). ELISA assays were performed on one donor in 2 separate repeat experiments (R1 and R2). All response measurements displayed have been corrected for background.

As shown in Fig 2 and Table 2, consistency between results produced by each operator improved considerably when assays were performed side-by-side in the same laboratory. As in round one pre-training assay comparisons, inter-site assay CVs were higher when the responses measured were weaker as seen with the more diluted supernatants for the ELISA assay (Fig 2A).

**Table 2:** IFNγ responses measured during “operator training” sessions.

Assay	Repeat/Donor	Antigen	Site 1	Site 2	Site 3	Mean response (SD)	CV (%)
ELISA (pg/ml)	Run 1	PPD (UD)	432	383.5	352	389 (40)	10
		PPD (1:3)	200	178	204	194 (14)	7
		PPD (1:9)	63	50	93.5	69 (22)	32
		PPD (1:27)	15.5	15.5	15.5	16 (0)	0
		PHA	402.5	402.5	407	404 (3)	1
	Run 2	PPD (UD)	140	133	118.5	131 (11)	8
		PPD (1:3)	31	38	35.5	35 (4)	10
		PPD (1:9)	15.5	15.5	21.25	17 (3)	19
		PPD (1:27)	15.5	15.5	34.5	22 (11)	50
		PHA	385	441	377.5	401 (35)	9
ELISpot (SFC)	1	FEC	290	256	301	282 (24)	8
		PPD	65	61	69	65 (4)	6
	2	FEC	9	6	8	8 (1)	17
		PPD	70	74	75	73 (2)	3
CD4 ICS (%IFNg+)		PPD	0.03	0.03	0.04	0.04 (0.01)	15
		FEC	5.73	5.51	5.65	5.63 (0.12)	2
CD8 ICS (%IFNg+)		PPD	0.92	1.07	0.94	0.98 (0.08)	8
		FEC	17.05	16.30	17.09	16.81 (0.45)	3

Operators from three sites performed three assays in a side-by-side fashion for the purposes of training

### Side-by-side operator training improves future across-site consistency of data for individually performed assays

**Fig 3:**
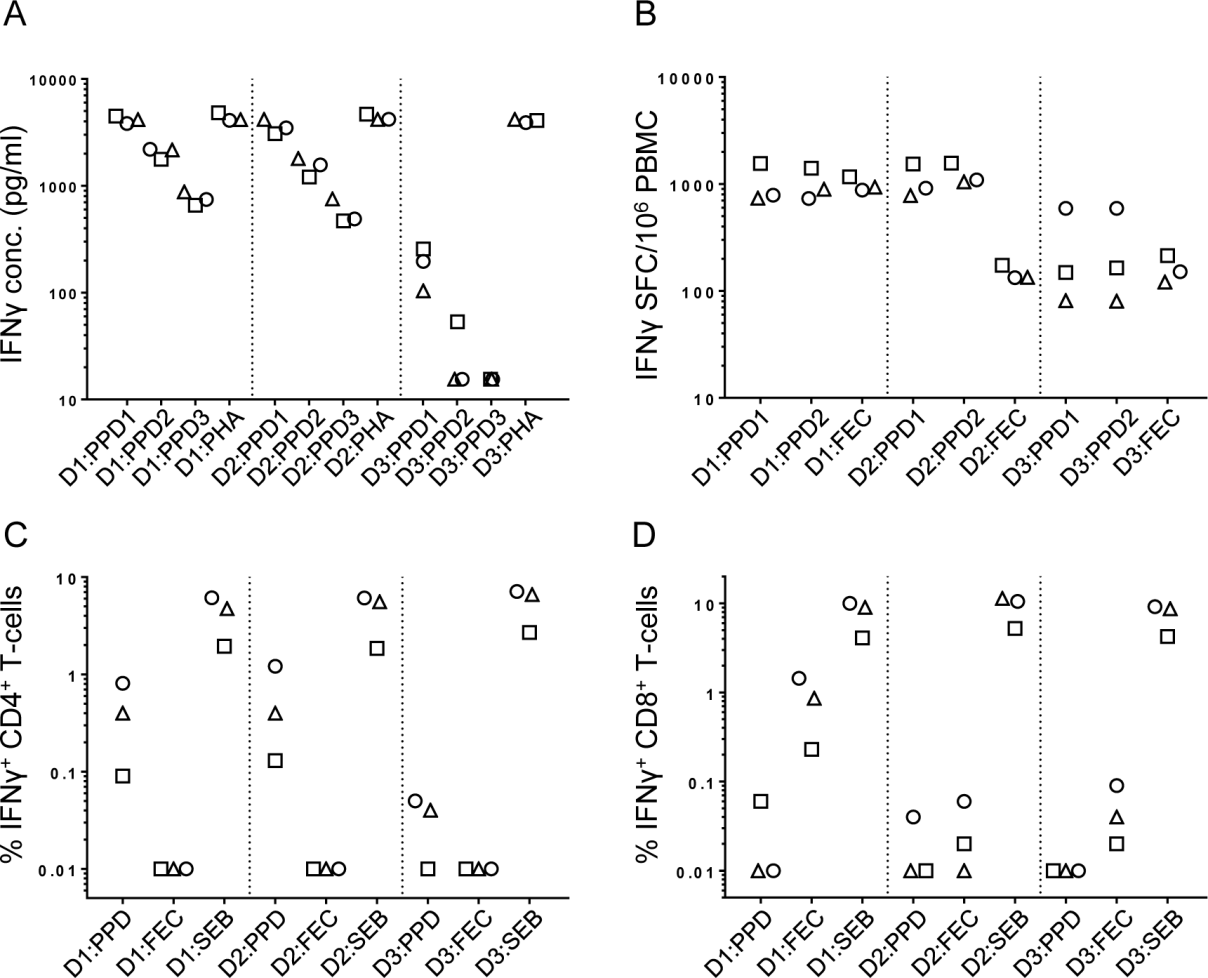
Post-training inter-site comparison of assay performance. Following joint inter-site training sessions, assays were repeated at the 3 participating sites using donors D1-3 as described for Fig 1. ELISA (A), ELISpot (B), CD4+ T-cell ICS (C) and CD8+ T-cell ICS (D); symbols indicate different sites: Site 1 (squares), Site 2 (triangles), Site 3 (circles). PPD supernatants were diluted for ELISA testing (PPD1-PPD3) as described for Fig 2. For the ELISpot assay, PPD stimulations were carried out on 2 different cell plating densities: PPD1, 3x10^6^ PBMC per well; PPD2, 1.5x10^6^ PBMC per well. All response measurements displayed have been corrected for background.

Following the side-by-side operator training sessions, each operator working individually at their own site repeated assays using aliquots of the same three donor PBMC described for round one experiments. Results of this round of experiments are shown in Fig 3 and Table 3.

**Table 3:** Post-training variation in IFNγ responses measured across three sites.

Assay	Donor	Antigen	Site 1	Site 2	Site 3	Mean response (SD)	CV (%)
ELISA (pg/ml)	A	PPD (UD)	4499	4169	3833	4167 (333)	8
		PPD (1:3)	1774	2171	2201	2048 (238)	12
		PPD (1:9)	659	879	747	762 (110)	14
		PHA	4829	4169	4107	4368 (400)	9
	B	PPD (UD)	3078	4183	3487	3582 (558)	16
		PPD (1:3)	1211	1800	1571	1527 (297)	19
		PPD (1:9)	470	755	490	572 (159)	28
		PHA	4688	4183	4194	4355 (289)	7
	C	PPD (UD)	257	104	197	186 (77)	41
		PPD (1:3)	54	16	16	28 (22)	78
		PPD (1:9)	16	16	16	16 (0)	0
		PHA	4096	4182	3914	4064 (137)	3
ELISpot (SFC)	A	PPD	1553	735	784	1024 (459)	45
		PPD (1:2)	1405	892	731	1009 (352)	35
		FEC	1169	933	875	992 (156)	16
	B	PPD	1540	779	912	1077 (406)	38
		PPD (1:2)	1567	1045	1089	1233 (290)	23
		FEC	174	134	133	147 (23)	16
	C	PPD	149	81	591	274 (277)	101
		PPD (1:2)	164	80	591	278 (274)	98
		FEC	214	121	151	162 (48)	29
CD4 ICS (%IFNγ+)	A	PPD	0.09	0.40	0.81	0.43 (0.36)	84
		FEC	0.01	0.01	0.01	0.01 (0)	12
		SEB	1.95	4.74	6.14	4.28 (2.13)	50
	B	PPD	0.13	0.40	1.21	0.58 (0.56)	97
		FEC	0.01	0.01	0.01	0.01 (0)	6
		SEB	1.85	5.57	6.12	4.51 (2.32)	51
	C	PPD	0.01	0.04	0.05	0.03 (0.021)	62
		FEC	0.01	0.01	0.01	0.01 (0.003)	23
		SEB	2.69	6.62	7.14	5.49 (2.43)	44
CD8 ICS (%IFNγ+)	A	PPD	0.06	0.01	0.01	0.03 (0.028)	101
		FEC	0.23	0.86	1.44	0.84 (0.61)	72
		SEB	4.10	9.01	10.00	7.7 (3.16)	41
	B	PPD	0.01	0.01	0.04	0.02 (0.02)	91
		FEC	0.02	0.01	0.06	0.03 (0.03)	96
		SEB	5.23	11.38	10.53	9.04 (3.33)	37
	C	PPD	0.01	0.01	0.01	0.01 (0)	0
		FEC	0.02	0.04	0.09	0.05 (0.04)	71
		SEB	4.25	8.70	9.20	7.38 (2.73)	37

Immune responses were tested using three assays, three blood donors and 3–4 antigens/positive control stimulants.

**Fig 4:**
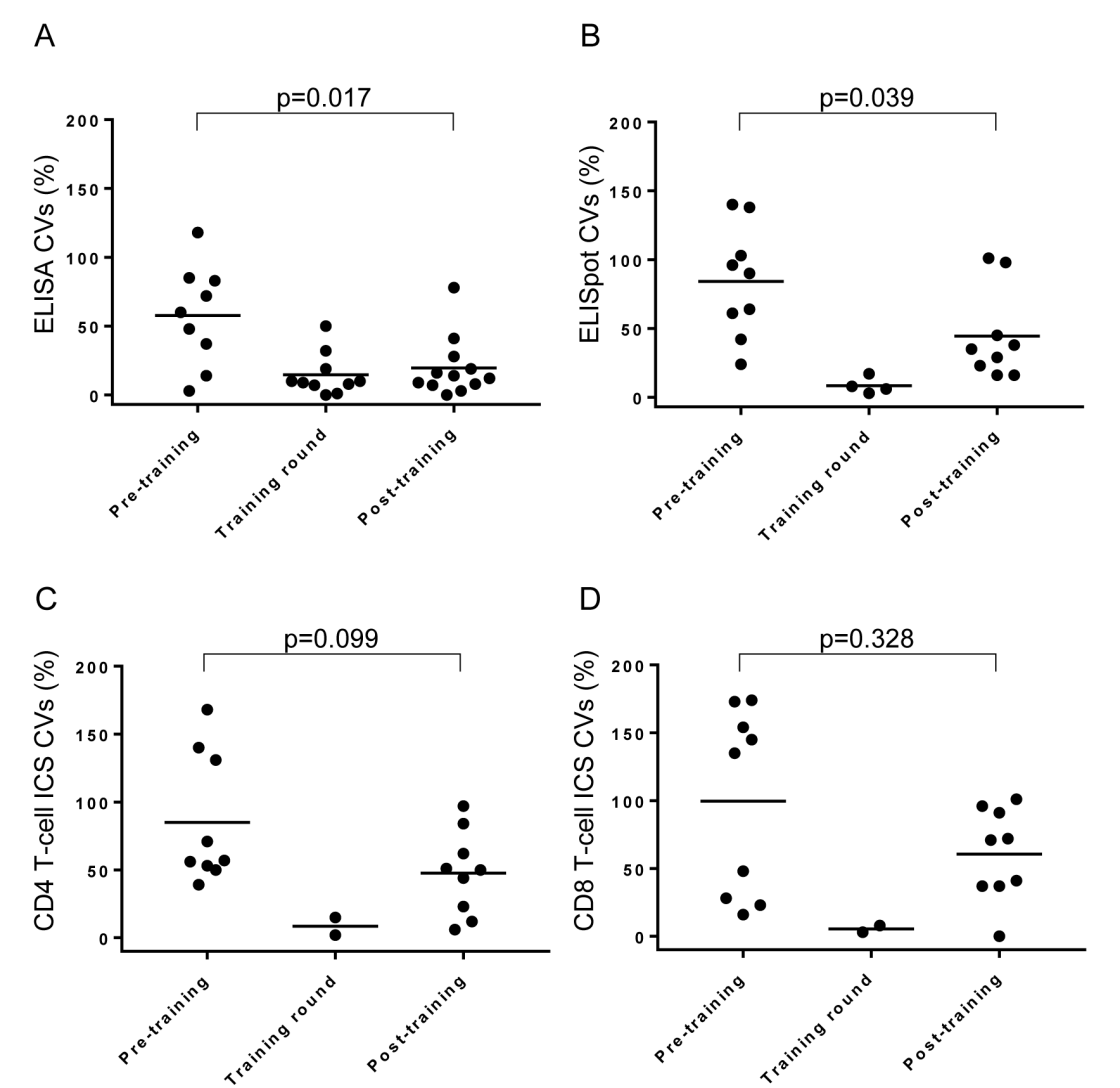
Side-by-side SOP training improves inter-site assay reproducibility. Inter-site coefficients of variance across all donors and stimulation conditions obtain both before, during and after side-by-side training were compared. Figure panels show comparisons for ELISA (A), ELISpot (B), CD4+ T-cell ICS (C) and CD8+ T-cell ICS (D). Bars indicate mean inter-site CV.

When all the inter-site CVs for different conditions (donor and stimulant combinations) are compared to those from previous rounds, a reduction in the mean inter-site CV as compared to the pre-training data was achieved for all assays (Fig 4). For the ELISA assay, the post-training data maintained the level of inter-site comparability seen for the training data and this was a significant improvement on the pre-training data (p = 0.017). Although the ELISpot and ICS assays did not manage to maintain the level of inter-site comparability seen when assays were performed side-by-side, a significant improvement in inter-site comparability between pre- and post-training data was seen for the ELISpot assay (p = 0.039). The improvements in CD4+ T cell and CD8+ T cell ICS assays post-training did not reach significance.

**Fig 5:**
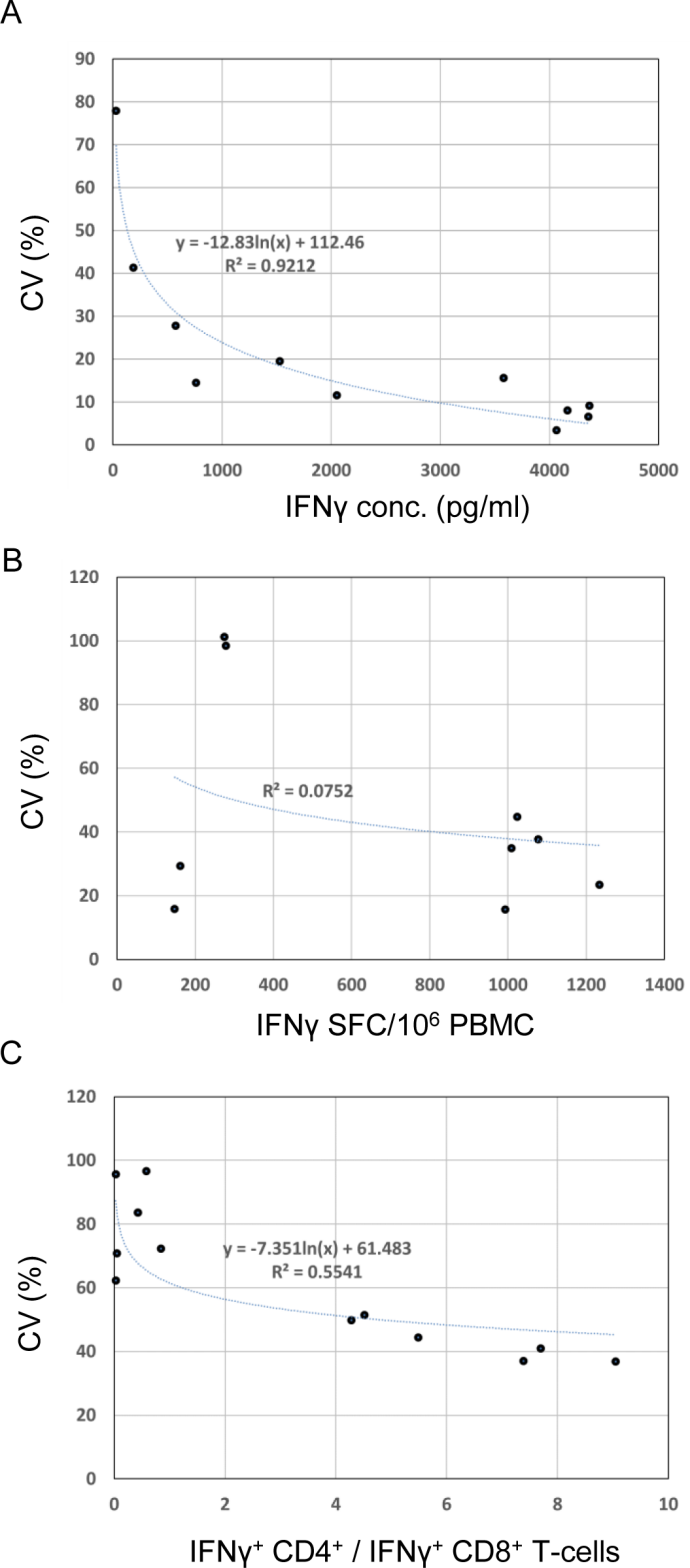
Lower magnitude responses are less readily measured consistently across sites for ELISA and ICS IFNγ assays. Inter-site mean assay responses were plotted against the corresponding inter-site CVs for ELISA (A), ELISPot (B) and ICS (C) assays. Data from CD4+ T-cell and CD8+ T-cell ICS assays were combined. Lines are logarithmic regressions with R^2^ values to indicate appropriateness of fit.

There was a trend for both the ELISA and ICS post-training datasets, although not for the ELISpot dataset, whereby the inter-laboratory CVs increased dramatically below a certain magnitude of measured response (Fig 5).

### TRANSVAC assay SOPs

Three detailed assay SOPs, produced as a result of the TRANSVAC project, are available online: http://www.transvac.org/SOPimmunoassays130627.pdf

## Discussion

Previous studies have addressed the issue of immune bioassay performance across multiple laboratories (including ELISpot, ICS and tetramer staining) and have highlighted the importance of removing protocol variations for improved inter-site comparability of data [17–20]. One of these studies also revealed that the number of years’ experience a particular laboratory group have with an assay, does not correlate with the ability of that laboratory to perform the assay better [17]. In agreement with that study, we also find here that guidelines and detailed protocols can only improve assay reproducibility across laboratories up to a point. We show that first hand observation and protocol training may achieve further improvement, even within experienced laboratories. It is easy to envisage how a group with many years’ experience of performing an assay might not appreciate the importance of external training of either long-standing or new personnel, when the necessary expertise exists “in-house”. However, the combined efforts of this and the studies referred to above demonstrate that this process should be deemed essential in inter-laboratory harmonisation, if multiple sites are to produce comparable data, irrespective of each site’s experience.

Even though the process of inter-laboratory training improved the comparability of data, some assays harmonized more readily than others did. Performance of the IFNγ ELISA assay post-training was as consistent across sites as it had been when assays were performed side-by-side and this was a significant improvement on inter-site comparability prior to training. Although ELISpot assay comparability also improved significantly following training, the post-training data was not as consistent across sites as the data produced during side-by-side assays, despite using the same spot counting equipment, software and count settings. There was a trend towards improved comparability post-training for both CD4+ T cell and CD8+ T cell ICS assays but this did not reach statistical significance. The performance variability of the ICS assay was also apparent in the less pronounced reduction in inter-site variability at higher response magnitudes. Although both the IFNγ ELISA and ICS assays showed a significant trend towards improved inter-site comparability at higher measured responses, the effect was stronger in the former assay where inter-site CVs were below 10% at the highest measurable responses. Even at higher measurable responses, the ICS assay did not result in inter-site CVs of greater than 40%. One of the most likely sources of additional variability for ICS assays is the flow cytometer instrument used, as this was the only parameter it was not possible to harmonise across each site.

We are not aware of any studies that take a systematic look at parallel technologies for a given biological measurement, identifying key parameters affecting concordance between and within laboratory groups. Others have described the in-depth qualification of individual assays similar to those we discuss in this manuscript [21,22], however this was not our aim here. Although the current study was relatively small, we have shown that parallel comparisons can reveal the critical aspects of each assay to focus on for better reproducibility. The use of standardised reagents, cells, reference materials and protocols, as well as staff expertise and training are critically important to understand the underlying diversity in outcomes from a given assay.

Although this study utilised experimental reagents and tools linked to tuberculosis vaccine research, the assays in question are equally applicable to diseases such as malaria and HIV. However, a cost-benefit analysis will be necessary as to whether money is best spent transporting all samples to a centralised analysis laboratory or on implementing the measures described here to ensure assay comparability across multiple laboratories. A centralised approach would ensure consistency in antigen and reagent usage, especially if the site in question was responsible for producing reference batches of these items. For example, following the current study, there was an interruption in the supply of PPD from Statens Serum Institute, however supplies of the same antigen from the UK National Institute for Biological Standards and Controls are available and induce equivalent assay responses.

Our conclusions, based on the present study, are that protocol optimisation and side-by-side training of operators may improve assay comparability across sites. However, the data suggest that below a certain threshold of response level, cellular assays become unavoidably variable in terms of responses measured. In addition, this effect appears to depend upon the assay in question. Assays that measure cellular responses directly, such as ICS, are more prone to variability. Assays such as the PBMC/IFNγ ELISA are less prone to such variations, probably as much of the assay takes a non-cellular detection approach (i.e. antibody pairs and a protein standard curve). These points should be borne in mind when selecting immuno-monitoring assays for use in vaccine clinical trials. The findings of this study may also inform the ongoing discussions on the strategies, investments and risks associated with centralising clinical immune studies to one lab versus decentralising and the potential for extracting GCP/GLP compliant data from multicentre studies.

## Supporting information

S1 Tables Background data as measured in unstimulated negative controls for ELISA, ELISpot and ICS assays run at each site. (DOCX)

## References

[1] Sow SO, Tapia MD, Diallo S, Keita MM, Sylla M, Onwuchekwa U, et al Haemophilus influenzae Type B conjugate vaccine introduction in Mali: impact on disease burden and serologic correlate of protection. Am J Trop Med Hyg. 2009;80: 1033–1038. 19478272

[2] Tameris MD, Hatherill M, Landry BS, Scriba TJ, Snowden MA, Lockhart S, et al Safety and efficacy of MVA85A, a new tuberculosis vaccine, in infants previously vaccinated with BCG: a randomised, placebo-controlled phase 2b trial. Lancet. 2013;381: 1021–1028. doi: 10.1016/S0140-6736(13)60177-4 23391465 10.1016/S0140-6736(13)60177-4 PMC5424647

[3] Shafer-Weaver K, Rosenberg S, Strobl S, Gregory Alvord W, Baseler M, Malyguine A. Application of the granzyme B ELISPOT assay for monitoring cancer vaccine trials. J Immunother. 2006;29: 328–335. doi: 10.1097/01.cji.0000203079.35612.c8 16699376 10.1097/01.cji.0000203079.35612.c8

[4] Streeck H, Frahm N, Walker BD. The role of IFN-gamma Elispot assay in HIV vaccine research. Nat Protoc. 2009;4: 461–469. doi: 10.1038/nprot.2009.7 19282851 10.1038/nprot.2009.7

[5] Donaldson MM, Kao S-F, Eslamizar L, Gee C, Koopman G, Lifton M, et al Optimization and qualification of an 8-color intracellular cytokine staining assay for quantifying T cell responses in rhesus macaques for pre-clinical vaccine studies. Journal of Immunological Methods. 2012;386: 10–21. doi: 10.1016/j.jim.2012.08.011 22955212 10.1016/j.jim.2012.08.011 PMC3646372

[6] Gómez CE, Perdiguero B, Cepeda MV, Mingorance L, García-Arriaza J, Vandermeeren A, et al High, broad, polyfunctional, and durable T cell immune responses induced in mice by a novel hepatitis C virus (HCV) vaccine candidate (MVA-HCV) based on modified vaccinia virus Ankara expressing the nearly full-length HCV genome. J Virol. 2013;87: 7282–7300. doi: 10.1128/JVI.03246-12 23596307 10.1128/JVI.03246-12 PMC3700320

[7] Abel B, Tameris M, Mansoor N, Gelderbloem S, Hughes J, Abrahams D, et al The novel tuberculosis vaccine, AERAS-402, induces robust and polyfunctional CD4+ and CD8+ T cells in adults. Am J Respir Crit Care Med. 2010;181: 1407–1417. doi: 10.1164/rccm.200910-1484OC 20167847 10.1164/rccm.200910-1484OC PMC2894413

[8] Akondy RS, Monson ND, Miller JD, Edupuganti S, Teuwen D, Wu H, et al The yellow fever virus vaccine induces a broad and polyfunctional human memory CD8+ T cell response. The Journal of Immunology. 2009;183: 7919–7930. doi: 10.4049/jimmunol.0803903 19933869 10.4049/jimmunol.0803903 PMC3374958

[9] Black GF, Weir RE, Floyd S, Bliss L, Warndorff DK, Crampin AC, et al BCG-induced increase in interferon-gamma response to mycobacterial antigens and efficacy of BCG vaccination in Malawi and the UK: two randomised controlled studies. Lancet. 2002;359: 1393–1401. doi: 10.1016/S0140-6736(02)08353-8 11978337 10.1016/S0140-6736(02)08353-8

[10] Lalor MK, Floyd S, Gorak-Stolinska P, Ben-Smith A, Weir RE, Smith SG, et al BCG vaccination induces different cytokine profiles following infant BCG vaccination in the UK and Malawi. J Infect Dis. 2011;204: 1075–1085. doi: 10.1093/infdis/jir515 21881123 10.1093/infdis/jir515 PMC3164434

[11] Geels MJ, Thøgersen RL, Guzman CA, Ho MM, Verreck F, Collin N, et al TRANSVAC research infrastructure—Results and lessons learned from the European network of vaccine research and development. Vaccine. 2015;33: 5481–5487. doi: 10.1016/j.vaccine.2015.01.079 25667962 10.1016/j.vaccine.2015.01.079

[12] Howles S, Guimarães-Walker A, Yang H, Hancock G, di Gleria K, Tarragona-Fiol T, et al Vaccination with a modified vaccinia virus Ankara (MVA)-vectored HIV-1 immunogen induces modest vector-specific T cell responses in human subjects. Vaccine. 2010;28: 7306–7312. doi: 10.1016/j.vaccine.2010.08.077 20816902 10.1016/j.vaccine.2010.08.077

[13] Spertini F, Audran R, Chakour R, Karoui O, Steiner-Monard V, Thierry A-C, et al Safety of human immunisation with a live-attenuated Mycobacterium tuberculosis vaccine: a randomised, double-blind, controlled phase I trial. Lancet Respir Med. 2015;3: 953–962. doi: 10.1016/S2213-2600(15)00435-X 26598141 10.1016/S2213-2600(15)00435-X

[14] Mensah VA, Gueye A, Ndiaye M, Edwards NJ, Wright D, Anagnostou NA, et al Safety, Immunogenicity and Efficacy of Prime-Boost Vaccination with ChAd63 and MVA Encoding ME-TRAP against Plasmodium falciparum Infection in Adults in Senegal. Richie TL, editor. PLoS ONE. 2016;11: e0167951 doi: 10.1371/journal.pone.0167951 27978537 10.1371/journal.pone.0167951 PMC5158312

[15] Black GF, Fine PEM, Warndorff DK, Floyd S, Weir RE, Blackwell JM, et al Relationship between IFN-gamma and skin test responsiveness to Mycobacterium tuberculosis PPD in healthy, non-BCG-vaccinated young adults in Northern Malawi. Int J Tuberc Lung Dis. 2001;5: 664–672. 11467373

[16] McNeil LK, Price L, Britten CM, Jaimes M, Maecker H, Odunsi K, et al A harmonized approach to intracellular cytokine staining gating: Results from an international multiconsortia proficiency panel conducted by the Cancer Immunotherapy Consortium (CIC/CRI). Cytometry A. 2013;83: 728–738. doi: 10.1002/cyto.a.22319 23788464 10.1002/cyto.a.22319 PMC4443815

[17] Britten CM, Gouttefangeas C, Welters MJP, Pawelec G, Koch S, Ottensmeier C, et al The CIMT-monitoring panel: a two-step approach to harmonize the enumeration of antigen-specific CD8+ T lymphocytes by structural and functional assays. Cancer Immunol Immunother. Springer-Verlag; 2008;57: 289–302. doi: 10.1007/s00262-007-0378-0 17721783 10.1007/s00262-007-0378-0 PMC2150627

[18] Smith SG, Joosten SA, Verscheure V, Pathan AA, McShane H, Ottenhoff THM, et al Identification of major factors influencing ELISpot-based monitoring of cellular responses to antigens from Mycobacterium tuberculosis. Neyrolles O, editor. PLoS ONE. 2009;4: e7972 doi: 10.1371/journal.pone.0007972 19956718 10.1371/journal.pone.0007972 PMC2776358

[19] Welters MJP, Gouttefangeas C, Ramwadhdoebe TH, Letsch A, Ottensmeier CH, Britten CM, et al Harmonization of the intracellular cytokine staining assay. Cancer Immunol Immunother. Springer-Verlag; 2012;61: 967–978. doi: 10.1007/s00262-012-1282-9 22714399 10.1007/s00262-012-1282-9 PMC3378841

[20] Smith SG, Smits K, Joosten SA, van Meijgaarden KE, Satti I, Fletcher HA, et al Intracellular Cytokine Staining and Flow Cytometry: Considerations for Application in Clinical Trials of Novel Tuberculosis Vaccines. Scriba TJ, editor. PLoS ONE. 2015;10: e0138042 doi: 10.1371/journal.pone.0138042 26367374 10.1371/journal.pone.0138042 PMC4569436

[21] Patton K, Aslam S, Lin J, Yu L, Lambert S, Dawes G, et al Enzyme-Linked Immunospot Assay for Detection of Human Respiratory Syncytial Virus F Protein-Specific Gamma Interferon-Producing T Cells. Clinical and Vaccine Immunology. 2014;21: 628–635. doi: 10.1128/CVI.00736-13 24574540 10.1128/CVI.00736-13 PMC4018879

[22] Kagina BM, Mansoor N, Kpamegan EP, Penn-Nicholson A, Nemes E, Smit E, et al Qualification of a whole blood intracellular cytokine staining assay to measure mycobacteria-specific CD4 and CD8 T cell immunity by flow cytometry. Journal of Immunological Methods. Elsevier B.V; 2015;417: 22–33. doi: 10.1016/j.jim.2014.12.003 25523923 10.1016/j.jim.2014.12.003 PMC4339399

