# Supplementary material for: Assay optimisation and technology transfer for multi-site immuno-monitoring in vaccine trials
Source: PLoS One. 2017 Oct 11;12(10):e0184391. doi: 10.1371/journal.pone.0184391 (PMC5636064; doi:10.1371/journal.pone.0184391)
Supplement: S1 Tables — (DOCX) [file pone.0184391.s001.docx]

**S1 Tables. Background data as measured in unstimulated negative controls for ELISA, ELISpot and ICS assays run at each site**

| Round | Donor | Site 1 | Site 2 | Site 3 |
| --- | --- | --- | --- | --- |
| Pre-training | 1 | 15.5 | 15.5 | 15.5 |
|  | 2 | 15.5 | 15.5 | 15.5 |
|  | 3 | 15.5 | 15.5 | 46 |
| Training round | L1 (run 1) | 15.5 | 15.5 | 15.5 |
|  | L1 (run 2) | 15.5 | 15.5 | 15.5 |
| Post-training | 1 | 55.8 | 15.5 | 68.2 |
|  | 2 | 37.8 | 15.5 | 15.5 |
|  | 3 | 78.7 | 15.5 | 90.7 |

ELISA responses in control (unstimulated) assays for each site (pg/ml)

| Round | Donor | Site 1 | Site 2 | Site 3 |
| --- | --- | --- | --- | --- |
| Pre-training | 1 | 109 | 54 | 10 |
|  | 2 | 32 | 21 | 9 |
|  | 3 | 19 | 11 | 7 |
| Training round | 1 | 4 | 4 | 4 |
|  | 2 | 6 | 6 | 5 |
| Post-training | 1 | 38 | 10 | 53 |
|  | 2 | 43 | 7 | 23 |
|  | 3 | 57 | 7 | 7 |

ELISpot responses in control (unstimulated) assays for each site (SFC per million PBMC)

| Response | Round | Donor | Site 1 | Site 2 | Site 3 |
| --- | --- | --- | --- | --- | --- |
| CD4 | Pre-training | 1 | 0.01 | 0.02 | 0.03 |
|  |  | 2 | 0.01 | 0.01 | 0.03 |
|  |  | 3 | 0.01 | 0.01 | 0.04 |
|  | Training round | 1 | 0.04 | 0.01 | 0.03 |
|  | Post-training | 1 | 0.06 | 0.05 | 0.01 |
|  |  | 2 | 0.06 | 0.03 | 0.01 |
|  |  | 3 | 0.05 | 0.02 | 0.04 |
| CD8 | Pre-training | 1 | 0.01 | 0.01 | 0.03 |
|  |  | 2 | 0.10 | 0.01 | 0.06 |
|  |  | 3 | 0.05 | 0.01 | 0.09 |
|  | Training round | 1 | 0.16 | 0.10 | 0.14 |
|  | Post-training | 1 | 0.17 | 0.04 | 0.01 |
|  |  | 2 | 0.04 | 0.06 | 0.04 |
|  |  | 3 | 0.06 | 0.05 | 0.06 |

ICS responses in control (unstimulated) assays for each site (%IFNγ+)
